# Divergence and convergence in international regulatory policies regarding genome-edited food: How to find a middle ground

**DOI:** 10.3389/fpls.2023.1105426

**Published:** 2023-01-30

**Authors:** Masashi Tachikawa, Makiko Matsuo

**Affiliations:** ^1^ Graduate School of Environmental Studies, Nagoya University, Nagoya, Japan; ^2^ Graduate School of Public Policy, The University of Tokyo, Tokyo, Japan

**Keywords:** genome-editing, regulation, policy convergence, divergence, trade

## Abstract

Regulations for organisms and products to which genome-editing technologies are applied are increasing in diversity, with the path-dependent effect of previous regulations for genetically modified organisms. Regulations for genome-editing technologies are a patchwork of international regulations that are difficult to harmonize. However, if the approaches are arranged in chronological order and the overall trend is examined, the regulation of genome-edited organisms and GM food products has recently been trending toward a middle ground which can be characterized as “limited convergence.” There is a trend toward the adoption of two approaches: one that considers GMOs but tries to apply simplified regulations and another that excludes them from the scope of regulations as non-GMOs but requires confirmation. In this paper, we discuss why there is a tendency toward convergence of these two approaches and examine the challenges and implications of these two approaches for the governance of the agricultural and food sectors.

## 1 Introduction

Regulations on living organisms and food products to which genome-editing technology is applied are being considered in various countries based on the current regulations on genetically modified organisms (hereafter GMOs). An overview of existing regulations (Menz et al., 2020; Entine et al., 2021; Turnbull et al., 2021; Jones et al., 2022) shows that only a patchwork of regulations exists instead of an international regulatory harmonization.

Regulatory review of genome-editing technology originally began with a review of existing regulations on GMOs in each country. This kind of review aimed to determine whether regulatory gaps existed between the new breeding techniques and existing regulations on GMOs. For example, in the European Union (EU), which was the earliest to examine the regulatory issues of new breeding techniques, including genome-editing technologies, the New Techniques Working Group was established within the European Commission in 2007 to identify and examine the characteristics of each technology and the regulations.

In identifying these regulatory gaps, each country is considering how to regulate organisms derived from genome-editing technologies, and some countries have responded by developing guidelines that partially revise or supplement existing laws regulating GMOs. International differences in the regulation of GMOs led to further differences in the regulation of organisms derived from genome-editing technology. This can be understood as path dependency in the sense that GMO policy has influenced subsequent policy on genome-editing technologies. As a result, international regulatory harmonization has become very difficult if not impossible. However, while regulatory approaches vary internationally, they are not randomly divergent. Rather, they converge in particular ways and within particular limits.

This study examines the regulatory considerations of various countries^1^, and confirms that a certain common direction, “limited convergence,” can be found in diversity. Then we discuss the regulatory context and reasons for this convergence. This study also discusses the socioeconomic implications of the lack of complete regulatory convergence, especially in the context of research and development, marketization, and trade. Genome-editing technologies are often discussed in three categories: SDN-1 (Site-Directed Nuclease 1), SDN-2, and SDN-3 (Podevin et al., 2013)^2^. SDN-3 is usually treated as subject to GMO regulations because it introduces foreign genes. Since the judgment on the treatment of SDN-1 and SDN-2 is different among countries, unless otherwise specified, the following discussion of genome-editing technologies will exclude SDN-3. The following section presents a framework for categorizing regulatory approaches in each country and then briefly reviews the regulatory situations of each country. Subsequently, the background to the convergence is examined as a cross-sectional discussion. Since complete convergence is unlikely to be reached, the socioeconomic implications of this situation are discussed, and conclusions are drawn.

## 2 Regulatory approaches and overview of each nation

Although many countries in the world are still considering the regulation of genome-edited organisms, some countries and regions have already established their own policies. At this point, regulatory approaches in each country can be broadly divided into the following four approaches (**Table 1** and **Box**).

**Table 1:** Four Approaches of Regulation of Genome-Edited Products.

Approaches	How the product is treated under the regulation: GMO or non-GMO	Applied Regulatory Oversight	Country or authority	
**Approach 1**	GMO	GMO Regulation as it is	EU, NZ (EPA)	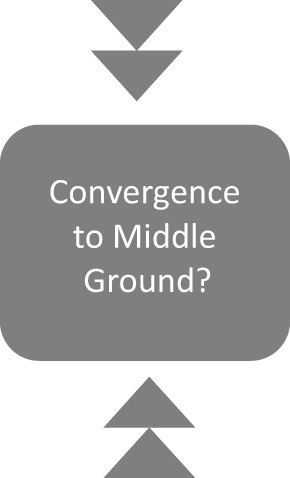
**Approach 2**	GMO	Simplified GMO regulations	UK*, FSANZ*, China	
**Approach 3**	non-GMO	Exempted but with confirmation by regulatory authority	Argentina and South America, Japan, India, Philippines	
**Approach 4**	non-GMO	Confirmation not required by regulatory authority	US (USDA), Australia (OGTR)	

An asterisk (*) indicates that it is under consideration. Since products using SDN-3 is subject to GMO regulations, SDN-3 is excluded from this Table.

Box **Explanation of the Four Approaches**

Approach 1: GMO regulations are applied as it is to genome-edited products. As a result, prior safety assessment and approval by the government are required.

Approach 2: Simplified GMO regulations will be applied to genome-edited products. As a result, simplified safety review and approval procedures will be applied.

Approach 3: Genome-edited products are exempt from GMO regulations. However, confirmation by the government is required before placing on the market.

Approach 4: Genome-edited products are exempt from GMO regulations. Prior confirmation is not required by the government.

The first position is to subject the genome-edited organisms to genetic modification regulations, which involves two approaches: (1) applying the GMO regulation as it is (Approach 1) and (2) applying a slightly simplified regulation (Approach 2). The former approach includes the EU and New Zealand Environmental Protection Agency, which considers genome-edited organisms to be the subjects to the same regulations as GMOs. The latter includes China and the United Kingdom (under discussion^3^), which are trying to adopt a framework that allows the authorization of genome-edited organisms through a simplified procedure in safety assessment while including them in the regulatory framework for GMOs.

The second position differs from the above position in that it excludes genome-edited organisms from the scope of GMO regulations. There are two approaches to this position: The first approach requires prior confirmation (Approach 3), while the second does not (Approach 4). The former approach, which includes Argentina and Japan, requires prior confirmation from the government, and if no problems are found after confirmation, the product is put on the market. Typically, if the developer notifies the government and the government (or the risk assessment agency) determines that it is not a genetically modified organism, it can be used commercially without any of the requirements stipulated for GMOs. The latter includes the US Department of Agriculture (hereafter USDA) and the Australian Office of Gene Technology Regulator (hereafter OGTR), where developers can make their own decisions and market products that are determined to be exempt from regulation without prior confirmation from the regulatory agency (allowing self-determination by developers).

Approaches 1 through 4 can be categorized as a sequence from the most to the least stringent in terms of regulation of organisms subject to genome-editing technologies. The difference between the above-mentioned two positions dictates whether the regulation of GMOs is focused on the technology applied (process-based regulation) or on the characteristics of the organisms produced (product-based regulation). Based on this repeatedly argued dichotomy, we note that a further difference is emerging within each position.

The following section provides an overview of the regulations of each country and describes their positions based on the above classification of approaches. **Table 2** shows when each country decided on their regulations based on such classification.

**Table 2:** Time Series of Regulatory Decisions of Genome-Edited Products in Each Country.

Approach	2014	2015	2016	2017	2018	2019	2020	2021	2022
Approach 1	New Zealand (EPA)				EU				
Approach 2								Australia & New Zealand (FSANZ)*	China (MARA), UK (Defra)*
Approach 3		Argentina		Chile Israel	Brazil	Japan			Canada (Health Canada), India, Philippines
Approach 4						Australia (OGTR)	US(USDA)		

An asterisk (*) indicates that it is under consideration.

### 2.1 The United States

The USDA, the Food and Drug Administration (hereafter FDA), and the United States Environmental Protection Agency (hereafter USEPA) have been regulating GMOs, and updates have been provided to the Coordinated Framework for Regulation of Biotechnology since 1986^4^. Each of the three agencies has been responsible for regulating transgenic organisms from its own perspective. The Office of Science and Technology Policy (OSTP) in the Executive Office of the President has played an important role in allocating and coordinating the roles of the three agencies. The decision to make genetically modified fish subject to FDA regulation is one example. Furthermore, the OSTP had urged the agencies to modernize their regulations to accommodate future biotechnology producsts. In this context, the USDA issued a notice in the Federal Register on May 18, 2020, regarding a major revision (the Sustainable, Ecological, Consistent, Uniform, Responsible, Efficient (SECURE) biotechnology regulations) to the biotech regulations (7 CFR 340)^5^. The SECURE rule clarifies the regulatory policy for plants with genome-editing applications. The USEPA also published a proposed review of the current Federal Insecticide, Fungicide, and Rodenticide Act (FIFRA) at the end of August 2020, and the FDA also collected comments in January 2017. Below is a summary of the regulatory policies of each ministry and agency.

#### 2.1.1 United States Department of Agriculture

The USDA-APHIS (Animal and Plant Health Inspection Service) has been regulating transgenic crops based on the Plant Protection Act from the viewpoint of regulating plant pests. In pre-SECURE regulations, developers sought safety review from the USDA under two procedures; notification and permit; those that were not found to pose a plant pest risk by the USDA were deregulated^6^. While building on previous regulatory experience, the revision of regulations was much needed to accommodate new technologies and new products. Against this backdrop, the USDA revised its federal regulation as the SECURE rule in May 2020, and through this update, the USDA has clarified its regulatory authority over plants derived from genome-editing technologies. They are now exempted from the regulation if any one of the following occur:

(1) The genetic modification is a change resulting from the cellular repair of a targeted DNA break in the absence of an externally provided repair template.
(2) The genetic modification is a targeted single base pair substitution.
(3) The genetic modification introduces a gene known to occur in the plant’s gene pool, or makes changes in a targeted sequence that correspond to a known allele of such a gene or a known structural variation present in the gene pool.
(4) The Administrator may propose to exempt plants with additional modifications, based on what could be achieved through conventional breeding.

As stated above, while template-based genome-editing technologies are subject to regulation, point mutations (to which oligonucleotide-directed mutagenesis (ODM) may fall under) are exempted from the regulation, unlike Australia and the Japanese Ministry of Environment. It is also important to note that the SECURE rule does not require an application to the USDA for a genetically modified (hereafter GM) crop that has been previously reviewed by the USDA, as long as a plant- trait- mechanism of action combination is the same. The limited scope of the regulation allows developers to self-determine^7^ and commercially grow GM crops that the USDA has previously screened and genome-edited crops as stipulated above^8^. The USDA allowed such self-determination to redirect the administrative resources to newly developed biotech products (NASEM, 2017) in the future.

#### 2.1.2 United States Environmental Protection Agency

The USEPA, under the FIFRA, has regulatory authority over pesticide ingredients produced in plants or plant-incorporated protectants (PIPs). This includes pesticide components such as cry1A, which is produced in Bt maize and other crops. In August 2020, the EPA released its proposed revisions to the FIFRA, which would exempt from the registration of PIPs, previously only allowed in conventionally bred plants, to plants derived from new technologies such as genome-editing technologies^9^. In other words, the proposal is to treat Bt crops produced by genome editing in the same way as crops obtained by conventional breeding. The USEPA also proposed that the Federal Food, Drug, and Cosmetic Act (FFDCA) allowable limits for PIPs be exempted for above-mentioned case. A final decision will be made based on public comment.

The proposed revisions would implement the Executive Order 13874, 84 Fed. Reg. 27899, “Modernizing the Regulatory Framework for Agricultural Biotechnology Products,” issued on June 11, 2019. This revision would advance the exemption from regulation for low-risk products, as required by the Executive Order.

#### 2.1.3 Food and Drug Administration

Under the “Statement of Policy: Foods Derived from New Plant Varieties” issued in 1992, the FDA accepts voluntary consultations from companies regarding GM foods and oversee safety on a case-by-case basis. This voluntary consultation system provides a comprehensive framework that can include new technologies such as genome-editing technologies in the sense that it covers “new plant varieties.” Therefore, the FDA did not introduce any new regulations; thus, the existing approach is still being used for genome-edited food.

The FDA regulates GM animals as well as GM foods under the FFDCA. GM animals are regulated as part of the animal drug regulations under the above law and are handled by the Office of Veterinary Medicine within the FDA. For animals derived from genome-editing technologies, the Draft Guidance for Industry on Intentional Modification of DNA in Animals (GFI #187) was published in January 2017. In contrast to the treatment of food products, this policy treats animals derived from genome-editing technologies as equivalent to GM animals and places them under strict regulation. The FDA’s policy has caused concern among companies and developers. In this context, in January 2021, the United States Department of Health and Human Services (the FDA’s headquarter) signed a memorandum of understanding with the USDA to consider changing the jurisdiction from the FDA to the USDA concerning livestock animals. However, while these developments are occurring, the FDA has decided to “not regulate” genome-edited slick-haired cattle through an exercise of enforcement discretion in March 2022^10^. The FDA has the option of taking this action on a case-by-case basis when an application is filed with the FDA and when there is little or no concern about trait alteration or food safety^11^.

Based on the above, the US regulation of genome-edited organisms falls under Approach 4 for USDA (crop) and EPA (under review), Approach 3 for FDA (food), and Approach 1 for FDA (animals) (under review). Different policies are applied to different subject areas in the United States due to each institutional jurisdiction. The existence of different policies for each item in the United States influence decisions within research and development and also has a significant impact on trade. These points are discussed later.

### 2.2 European Union

In the EU, the Environmental Release Directive was revised in 2001 (Directive 2001/18/EC). The revised directive incorporated the precautionary principle, reflecting public concerns about food safety and new science/technology at the time, such as the bovine spongiform encephalopathy (BSE) scandal. In this directive, GMOs are defined as those to which specific technologies (those listed in Annex 1A, Part 1) were applied: “genetically modified organism (GMO) means an organism, with the exception of human beings, in which the genetic material has been altered in a way that does not occur naturally by mating and/or natural recombination” (Article 2). Among mutagenesis technologies, those that do not use recombinant nucleic acid molecules or GMOs were categorized as GM techniques but were excluded from the Directive (Annex 1B). This was because they “have conventionally been used in a number of applications and have a long safety record” (“whereas clause” (17) of the Directive). In 2015, the Directive was further amended to permit EU member states to prohibit the cultivation of GM crops in their territory based on environmental or agricultural policy objectives. Against this backdrop, new breeding techniques have been attracting attention.

Since around 2007, the EU has been considering how to deal with new breeding techniques, which are difficult to position under the existing GM regulations. The European Commission established the New Breeding Techniques Working Group to examine the regulatory status of new technologies, including other technologies that already existed at that time. The European Joint Research Center also examined eight new technologies (e.g., zinc finger nucleases and reverse breeding) and their relationship to the regulations (Lusser et al., 2011). As the results of these studies were disseminated, industry associations, such as EuropaBio, the European Seed Association, and anti-GM campaign groups tried to express their own views to influence the EU policy.

Although various positions on the potential of the new breeding technology and its regulatory status were discussed, several years passed without a clear regulatory policy from the European Commission. In the meantime, environmental groups filed a lawsuit against the French government over the legal status of mutation breeding. This then led to the problem of the legal status of genome-editing technology, and the French court asked the Court of Justice of the European Union (CJEU) for a legal interpretation of mutagenesis technology, including genome-editing technology. As a result, in July 2018, the CJEU rendered a decision regarding the legal status of products derived from mutagenesis under the Environmental Release Directive (Court of Justice of the European Union, 2018). In conclusion, it was held that organisms derived from mutagenesis are, in principle, GMOs and are subject to the legal obligations of the Directive. However, those with a long history of safe use (e.g., radiation breeding) were excluded from the scope of the regulation in accordance with the provisions of Annex 1B of the Directive. In other words, organisms derived from genome-editing technologies without a long history of safe use were deemed to be GMOs and subject to the regulation under the EU’s Environmental Release Directive.

The ruling of the CJEU had a significant impact on European stakeholders, as it affected the position of genome-editing technologies in the EU as a whole. In response to this ruling, the European Commission was instructed to gather information regarding new genomic techniques (e.g., regulatory status in member states, detection techniques, risk assessment, market trends, ethical considerations, etc.) from various EU institutions. The results were compiled in April 2021. In September 2021, the Commission then presented a roadmap for the future for public comment to consider a legal framework for targeted mutagenesis and cisgenesis in plants. The draft regulation is expected to be published in 2023. The United Kingdom, which has left the EU, has begun to consider its own regulations as the Precision Breeding Bill.

Based on the above, the European Union’s position at this point represents Approach 1.

### 2.3 Argentina and other South American countries

South American countries, especially Argentina, Brazil, Chile, Paraguay, and Colombia, are clarifying their regulatory positions and starting commercialization from the standpoint of promoting genome-editing technology (Kuiken and Kuzma, 2021). The following discussion is limited to Argentina as a representative country^12^.

In May 2015, Argentina’s Ministry of Agriculture, Livestock, and Fisheries (MAGyP) established a “prior consultation procedure” for crops derived from new breeding techniques such as genome-editing technologies (Decision 173/15). This decision was the first of its kind in the world and was subsequently followed by other South American countries. In the pre-consultation process, the product is examined to determine whether “novel combination of genetic material” (foreign genes) remain in the genome (Whelan and Lema, 2015). The Ministry of Agriculture, Livestock and Fisheries accepts the preliminary consultation and then asks the Committee on Biosafety (CONABIA) to review it and decide whether it should be subject to regulation. This includes instances of transient introduction; if the foreign gene does not remain in the final organism, it will not be subject to regulation.

However, even if the crop is treated as a non-GMO, the relevant government department will consider additional measures if the novelty of the crop exists and such measures are deemed necessary. This Argentine approach of making regulatory decisions based on the presence or absence of foreign genes is also being adopted by neighboring countries. The results of this prior consultation are not made public^13^. This is because it is believed that public disclosure would distinguish certain technologies from conventional breeding and could lead to discriminatory treatment.

From the above, the current position of Argentina and other South American countries represents Approach 3, although there are some minor differences within the region.

### 2.4 Oceania

#### 2.4.1 Australia

In Australia, the environmental safety of GMOs is regulated by the OGTR under the Gene Technology Act of 2000 and the Gene Technology Regulations enacted in 2001. The OGTR oversees the implementation of regulations.

In light of the emergence of new breeding techniques such as genome-editing technologies, the OGTR, after several years of study, revised their regulations on April 4, 2019, that is, the Gene Technology Amendment Regulations 2019. The revision clarifies that genome-editing technologies that fall under SDN-1 are exempted from the current regulation, while genome-editing technologies that use artificially created templates outside the cell (SDN-2) are subject to the regulation^14^.

The revisions made in 2019 include the following points:

1) Revisions to Schedule 1A (technologies that are not gene technologies) to clarify the conditions under which RNA transfection is not considered a gene technology.

2) Revisions to Schedule 1 (organisms that are not GMOs) to add six items, including the case without templates and matters related to null segregants.

3) Establishment of Schedule 1B (technologies that are gene technologies) to clarify technologies that use ODM and templates.

Although the revisions exclude some genome-editing technologies (SDN-1) from the scope of the regulation, its content can be considered to be identical as the USDA’s regulations and the Japanese Ministry of Environment’s policy (see below) in terms of making genome-edited organisms that use templates being subject to regulation. In addition, the OGTR rules share with the USDA rules in that no confirmation is required for exemptions from the regulations and developers can self-determine.

#### 2.4.2 New Zealand

In New Zealand, genome-edited organisms are subject to GMO regulation, and like in the European Union, this was triggered by a court case. In April 2013, the New Zealand Environmental Protection Agency (hereafter NZEPA) ruled that trees produced with ZFN-1 (Zinc Finger Nucleases) and TALEN (Transcription Activator-Like Effector Nucleases) were not subject to regulation under the country’s GMO control law, the Hazardous Substances and New Organisms Act (HSNO Act). However, environmental NGOs appealed this administrative decision and filed a lawsuit. Then, in May 2014, the High Court ruled, and the arguments of the plaintiff NGOs were accepted ((New Zealand High Court Wellington Registry, 2014)), stating that ZFN-1 and TALEN should not be excluded from HSNO Act. According to the High Court, these genome-editing technologies were considered novel and not scientifically well-established. Therefore, in view of the precautionary approach on which the HSNO Act relies, the judge ruled it as inappropriate to exclude these techniques from the HSNO Act.

In response to the ruling, the NZEPA revised the relevant statutes to explicitly state that mutagenesis techniques utilized before July 29, 1998 (the effective date of the HSNO Act) would be treated as non-GMOs. In other words, those created using mutagenesis technologies, including genome-editing technologies, developed after July 1998 are now subject to regulation as GMOs.

#### 2.4.3 Food Standards Agency of Australia and New Zealand

While environmental safety is regulated by Australia’s OGTR and the NZEPA, food regulations are regulated by the Food Standards Agency of Australia and New Zealand (FSANZ), which has been established jointly by the two countries. In particular, the revision of Food Standards, which define GM foods, is an issue. In February 2018, the FSANZ collected opinions on the revision, compiled the results in December 2019, and published a draft proposal (P1055) of the Food Standards in October 2021^15^. In the proposal, FSANZ indicated to expand the process-based definition to capture all methods for genetic modification including genome-editing. However, at the same time, FSANZ is proposing to revise the definition for ‘food produced using gene technology’ to exempt certain products based on product-based criteria. Criteria includes food from which foreign genes have been removed, food with characteristics that can be produced by conventional breeding, and processed food that does not contain foreign genes or new proteins. Food that does not meet these exclusion criteria will be subject to a prior safety review. A final decision is expected to be made after further review.

From the above, Australia’s OGTR represents Approach 4, NZEPA represents Approach 1, and FSANZ (under review) represents Approach 2.

### 2.5 Asian countries

After 2019, Asian countries have also been actively considering regulations. The following sections discuss Japan, China, India, and the Philippines as countries that have clarified their regulatory policies.

#### 2.5.1 Japan

In Japan, the Ministry of the Environment (MOE) decided in February 2019 and the Ministry of Health, Labor and Welfare of Japan (MHLW) in September of the same year on the policy for handling genome-edited organisms from the context of environmental safety and food safety, respectively (Matsuo and Tachikawa, 2022). The MOE’s decision was a Director-General’s notice, while the MHLW’s was a decision by a counselor, and the handling policy was determined by administrative decision without revision of laws and regulations. The policy of both ministries state that genome-edited organisms are exempted from the regulation of GMOs, but there are some differences between the policies of the two ministries.

According to the MOE, those not containing foreign genes are exempted from the regulation, but those created using templates such as SDN-2 are subject to the regulation. On the other hand, according to the MHLW, those with “a risk that could occur even with conventional breeding techniques” were exempted from the regulation. Therefore, based on the MHLW, SDN-1 is not regulated, and SDN-2 is judged on a case-by-case basis. In Japan, even when exempt from regulations, confirmation by each administrative body is required and labeling is encouraged by the government.

The Japanese regulations do not distinguish between plant, animal, and microorganism, and the rules have been clarified for all types of uses, such as cultivation, food use, and feed use.

#### 2.5.2 China

In January 2022, the Ministry of Agriculture and Rural Affiars (MARA) of the People’s Republic of China published the “Guidelines for Safety Evaluation of Gene-Edited Plants for Agricultural Use (trial)”^16^. If the risk is found to be low, a small-scale intermediate test would be conducted, and the results are submitted to apply for a safety certificate for commercial production. The above guidelines have been adopted by the Chinese government.

Therefore, the above guidelines attempt to promote research and development and commercial use by maintaining China’s existing GM regulations and including plants derived from genome-editing technologies. This introduces a simplified procedure (i.e., a safety certificate can be applied for after a small-scale test). While the basic legal regime for GMOs in China has been maintained, China has also been actively revising its regulations on GM crops in recent years and trying to promote the use of life sciences^17^.

#### 2.5.3 India

On March 30, 2022, the Ministry of Environment, Forest, and Climate Change of India issued an office memorandum^18^ and decided that plants produced under SDN-1 and SDN-2 that do not contain foreign genes are not subject to GM regulations. In other words, the Ministry has indicated that Articles 7 to 11 (import/export, manufacturing/processing, environmental release, food use, etc.) of the GM Regulations (Regulations for the Manufacture, Use/Import/Export and Storage of Hazardous Microorganisms/Genetically Engineered Organisms or Cells, 1989) are exempted for those plants.

In May 2022, the Department of Biotechnology, Ministry of Science and Technology also released the “Guidelines for Risk Assessment of Genome-Edited Plants”^19^ to provide information on where applicants can submit notifications and detailed application procedures.

#### 2.5.4 Philippines

The Philippines has been conducting technical and regulatory studies on new plant breeding technologies since 2016. In particular, since June 2019, a decision has been made by the Philippine government to develop a government policy under the Department of Agriculture, and guidelines on plant breeding innovations (PBI) have been considered. In May 2022, the Philippine Department of Agriculture issued Memorandum Circular No. 8 based on the above considerations and published the rules and procedures for the marketing of products based on PBI^20^. Products that do not contain exogenous genes (new combinations of genetic material) were exempted from the regulations. In particular, developers were to provide information and follow procedures to the Bureau of Plant Industry of the Department of Agriculture. If the organism is exempted from the GM regulations (JDC1), a certificate is issued to the developer, and the information excluding confidential information is published on the website.

Therefore, it is clear that in Asia, China reflects Approach 2 and Japan, India, and the Philippines have reflected Approach 3. What approach other Asian countries will adopt still needs to be examined.

## 3 Discussion: Cross-regulatory considerations

Various approaches have been adopted by multiple countries. However, if the approaches are arranged in chronological order and the overall trend is examined (**Table 2**), it can be seen that the regulation of genome-edited organisms has recently been trending toward a middle ground. In other words, there seems to be a trend toward the adoption of two approaches: one that regards them as GMOs but tries to apply simplified regulations (Approach 2) and one that excludes them from the scope of regulations as non-GMOs but requires confirmation (Approach 3). These two approaches are an attempt to take a middle ground between applying strict GMO regulations and excluding GMOs from the regulations as equivalent to conventional breeding. The following sections discuss why there is a tendency at this stage to converge on these two approaches and examine the challenges and implications of these approaches for the governance of the agricultural and food sectors with the application of genome-editing.

### 3.1 Why convergence is emerging

Although further considering the regulatory situation in each country is essential, hypothesizing a couple of points regarding why convergence is currently occurring is possible^21^. This study will discuss (1) the background to the limited number of jurisdictions adopting Approaches 1 and 4 and (2) the background to the convergence toward a middle ground approach.

The EU and New Zealand are the only jurisdictions that are currently adopting the same regulations to organisms subject to genome-editing technologies as to genetic modification (Approach 1)^22^. However, the current policies of the EU and New Zealand were decided through court processes rather than voluntarily adopted by their respective governments. Furthermore, the laws and regulations referred to by the judges in the EU and New Zealand were all enacted around the year 2000 (the Environmental Release Directive 2001/18/EC in the EU and the HSNO Act 1996 in New Zealand.), when concerns about science, technology, and the precautionary principles were being emphasized in the wake of the BSE incident in Europe. At this point, it is unknown whether there will be more countries adopting Approach 1 in the future. However, given the high expectations of industry and governments for genome-editing technologies, it is not highly likely that such an approach will be widely adopted.

Second, contrary to the above, only the Australian OGTR and the USDA adopt Approach 4 and allow the use of genome-edited organisms without confirmation from the government. However, both of these agencies authorize organisms only for environmental release, and another government agency will be involved in the regulation of food use, such as FDA and FSANZ. The Australian OGTR found it difficult to introduce a notification system because “organisms modified using SDN-1 [ … ] do not pose risks that warrant regulating these organisms as GMOs” and regard them out of regulatory scope (OGTR, 2018, p.25). Conversely, the USDA attempted to strike a balance with genome-edited organisms by excluding GM crops that met certain conditions outside the scope of the regulation *via* the recently mentioned regulatory revision. Therefore, the number of countries adopting Approach 1 and 4 has been limited to date.

Considering the above, we would like to discuss the underlying factors of the convergence phenomenon currently occurring. Policy convergence and policy transfer have been discussed in policy studies (Bennett, 1991; Vogel and Kagan, 2004; Holzinger and Knill, 2005) regarding the underlying factors of international policies moving toward the same content and direction. In the context of globalization, policy harmonization has been promoted under the leadership of international organizations (e.g., the World Trade Organization) and/or leading nations in various fields (e.g., the US and the EU).

To date, however, there has been no clear policy coordination effort on how to regulate genome-edited organisms in international organizations such as the Cartagena Protocol of Biosafety (CPB), the Codex Alimentarius Commission (Codex), and the Organization for Economic Co-operation and Development (OECD)^23^. Policy formation has been conducted in the absence of clear rules, with each country accumulating its own domestic considerations and information gathering. In this sense, a phenomenon that could be discussed by policy convergence theory, rather than the policy transfer theory, which focuses is on the process by which a particular policy model diffuses (Knill, 2005), has emerged. In the theory of policy convergence, the focus is on the effects of the commonality of the situation in which countries find themselves, resulting in policy convergence. Therefore, it is appropriate to focus on this theory in this study^24^.

The policy convergence theory proposes that there are two types of factors that facilitate convergence: (1) causal mechanisms and (2) facilitating factors (Knill, 2005). The former includes independent problem-solving, international harmonization, regulatory competition, and transnational communication. The latter includes cultural, institutional, and socioeconomic similarity. Based on the findings of these previous studies, there are three relevant points to consider regarding the convergence of regulations on genome-edited organisms.

First, while there are high expectations for the potential of genome-editing technologies, countries are not only engaged in research and development but also regulatory competition (Knill, 2005). Several countries have developed strategies and policy documents that seek to increase industrial competitiveness while maximizing the use of genome-editing technologies (e.g., the US Agricultural Innovation Strategy and Japan’s Biotechnology Strategy). These expectations for new technologies (Borup et al., 2006; Yamaguchi and Fukushima, 2019) are likely to increase the tendency to adopt a more technology-promoting orientation due to the international presence of regulatory competition. In this situation, Approach 1 will be less likely to be adopted.

Second, and related to the above, it is conceivable that countries have rushed to enact rules through administrative procedures alone rather than by revising basic laws due to the extreme regulatory competition. When a political review process is introduced, seeking a compromise that transcends political positions is essential, and a variety of options will be explored. Opportunities for participation in policy formation processes from civil society organizations will also increase, and involvement from groups that have demonstrated opposition to GMOs may adopt Approach 1. Which approach will be chosen will be unpredictable. To avoid such risks, convergence may have resulted from the fact that most countries have tried adopting regulations through administrative procedures rather than legal revisions. This point is also related to the next aspect of institutional similarity.

Third, the process of determining regulations for the handling of genome-edited organisms has been handled by the administrative bodies in charge of regulating GMOs in each country. These administrative agencies have overseen food safety, environmental safety, and other regulatory enforcement, as well as external information gathering through regulating GMOs. In the domestic context, these agencies have consultative processes with relevant experts and stakeholders regarding safety and proper handling and have continued to exchange information with various international forums such as the CPB, and the Codex, and the OECD. These administrative procedures would be considered to have enhanced their institutional similarity as far as scientific information and stakeholder opinion is collected as typical procedures for authorization^25^. In addition, since the emergence of new breeding technologies, including genome-editing technologies, this topic has been discussed at various international meetings, such as the OECD and the APEC, regarding their opportunities and challenges. In this context, the institutional similarities and transnational communication highlighted by Knill (2005) may have encouraged policy convergence regarding genome-edited organisms.

**Table 3** summarizes the above points as the main actors involved in the formation of regulations.

**Table 3:** Actors Involved with Introduction of Regulatory Measures related to Genome-Edited Organisms.

Approach	Country	Decisions/Measures	Administrative Process	Parliamentary Process	Court Ruling
1	EU/France	Court of Justice of the European Union (2018)	⚫		⚫
1	New Zealand	High Court Decision (2015)	⚫		⚫
2	China	Guidelines for Safety Evaluation of Gene-Edited Plants for Agricultural Use (Trial) (2022)	⚫		
2	Australia&NZ (FSANZ)	Foods Standard Codes: Proposal P1055 (2021)	⚫		
2	UK	(Draft) Precision Breeding Bill	⚫	⚫	
3	Argentina	Resolution No. 173/2015 (2015)	⚫		
3	Japan	Decision of MOE and MHLW (2019)	⚫		
3	India	MOEFCC Office Memorandum (2022)	⚫		
3	Philippines	MOA Memorandum Circular No. 8 (2022)	⚫		
4	US(USDA)	SECURE rule (2020)	⚫		
4	Australia (OGTR)	Gene Technology Amendment Regulations 2019 (2019)	⚫	⚫(1)	

(1): In Australia, changes to the Gene Technology Regulations are tabled in Parliament for 15 days and if no objections are raised, the changes are implemented as proposed.

### 3.2 Limited negative responses

Why has the approach of revising the law not been taken? This may be related to the fact that there have not been much negative responses against genome-edited food. Two factors may be involved in this situation: (1) social recognition of genome-edited food has been suppressed by various factors and (2) the EU has imposed regulations same as those for GMOs.

First, the lack of major negative responses of genome-edited food is related to the fact that genome-editing has been used for multiple purposes, in particular, for medical applications and, as a result, has attracted much attention in the medical field. For example, while the birth of genome-edited babies in China (Wang et al., 2019) led to an instant increase in social recognition of this technology, its application to agriculture and food products has not attracted comparable social attention (Shew et al., 2018; Gatica-Arias et al., 2019; McFadden et al., 2021). In addition, in countries where commercial release has already begun, the following measures are also presumably linked to the result that awareness among general consumers is not high (Kato-Nitta et al., 2021). In other words, it is difficult for general consumers to be aware of the results of the confirmation of genome-edited food because they are not disclosed, as is the case in Argentina. In countries where information is disclosed, such has in the United States and Japan, the food is distributed for commercial use or direct to consumers (D2C) and not for general market distribution, and this discouraged the expression of concern by general consumers or retailers. These factors may explain the lack of public awareness of genome-edited food.

Second, the lack of negative responses may be related to the actions of civil society organizations in the EU, which have been very vocal in their opposition to GM food. In other words, it may be related to the fact that the Europe Union currently regulates foods with genome-editing as GM food. In this context, the movement within the EU has not gained much momentum. A significant resistance to GMOs has occurred in the EU since 1996, when GM soy was criticized for being imported without labeling (Schurman and Munro, 2006). Unlike in the United States, the public protest movement in the EU is characterized by its high visibility and open political campaigns (Bernauer and Meins, 2003)^26^. If there is a move to revise the regulations in the EU in the future, a wave of criticisms regarding genome-editing originating in the EU may occur^27^.

### 3.3 Challenges posed by the two approaches

As far as the countries considered in this paper are concerned, the regulation of genome-editing is positioned by most countries to be somewhere between the situation of no regulation at all and the situation of strict regulation equivalent to that of GM. However, in a strict sense, policy convergence has not yet been fully reached. The difference in regulatory status between GM (Approach 2) and non-GM (Approach 3) results in significant differences, especially when ex-post facto regulations such as GM labeling, traceability, crop registration, and license renewal are applied. On the other hand, if these ex-post facto regulations are exempted, there may not be a significant difference between GM (Approach 2) and non-GM (Approach 3). What are the implications of such regulatory measures for the introduction of genome-edited foods into society? Although there are only a limited number of cases in the market so far, it would be beneficial for countries currently considering regulations to consider these issues and implications.

#### 3.3.1 Issues in research and development

In the research and development stage, which is the stage prior to receiving confirmation from the regulatory authorities, it is expected that even if an organism is ultimately exempted from the regulation as an organism derived from genome-editing technologies, it will be treated as a GMO subject to biosafety regulations. However, if there are significant differences between Approach 2 and 3 in the subsequent safety review procedures, there may be substantial differences in research and development. As a result, there would be a significant disparity in the application process for developers. In Argentina, where Approach 3 has been adopted, the percentage of domestic companies developing genome-edited products has increased compared to that of GMOs (Whelan et al., 2020). Depending on the size of the burden of safety review procedures, there could be a significant disparity in the number and types of products developed. If the burden is lessened, it is possible that a greater variety of research and development processes could be pursued with more diverse development goals.

#### 3.3.2 Issues in marketization

In the case of Approach 1 and Approach 2, GM labeling regime raises concerns from consumers, which will make the commercialization of the product difficult. There is a possibility that genome-editing will be applied only to products that are not subject to mandatory labeling or non-food uses (e.g., flowers).

On the other hand, if genome-edited organisms are exempted from the GM regulation as in the case of Approach 3 and Approach 4, they will not be labeled, which will encourage their use in a variety of products. Since labeling is not required for such products, the number of products with genome-editing is expected to steadily increase.

Existing studies indicate that consumers have the same concerns about genome-editing technology as they do about transgenic technology, while there are different concerns regarding the purpose of the technology’s application and the organisms (plants or animals) to which it is applied (Kato-Nitta et al., 2021; Busch et al., 2022). Regarding concerns about the technology itself, some argue that there is no need to distinguish between genome-editing technology and genetic modification technology (Mikami and Tachikawa, 2019). From this standpoint, criticism may be directed at the invisible distribution of products based on genome-editing within markets. In this context, there is a possibility that distribution through commercial use or D2C, rather than general market distribution, will continue in the future.

#### 3.3.3 Issues concerning trade

The approaches that different countries adopt will also pose significant challenges for trade. There are several challenges, but this study focuses on three points as follows.

The first is the asymmetry problem that arises when trading partners take different approaches. Based on **Table 4**, when genome-edited food is exported from countries that adopt Approach 3 (confirmation is required) but do not disclose notification results (e.g., Argentina) or from countries that adopt Approach 4 (companies are allowed to make their own decisions) to countries that adopt Approach 1 or 2, if the exporters do not actively provide information, concerns regarding GM food being distributed under cover (“hidden GMOs”) may spread which disrupts the market (Bertheau, 2021). Some consumer groups are demanding that developers of genome-edited products should develop tracking methods and ensure consumers’ right to choose^28^. In short, the issues of information disclosure and transparency from exporter to importer are important. In light of the major opposition which happened in Europe over the importation of GM soybeans in 1996, it is undeniable that the same thing could occur with genome-edited products, and negative reactions could grow in the future.

**Table 4:** Expected Response of Importing Country in Different Situations.

		Importing Country			
		Approach 1	Approach 2	Approach 3	Approach 4
Exporting Country	Approach 1	Under GMO regulations	Under GMO regulations	Consumer Negative Responses	Consumer Negative Responses
	Approach 2	Under GMO regulations	Under GMO regulations	Consumer Negative Responses	Consumer Negative Responses
	Approach 3	Advance Notice/Pre-market Authorization	Advance Notice/Pre-market Authorization	Advance Notice/Pre-market Authorization	Conventional Trade
	Approach 4	“Hidden GMO”	“Hidden GMO”	Advance Notice/Pre-market Authorization	Conventional Trade

If a country with Approach 3 does not disclose genome- editing information to traders, similar responses in the case of Approach 4 would happen.

Exports from countries adopting Approaches 1 or 2 to countries adopting Approaches 3 or 4 may be avoided by consumers in the importing country because the genome-edited product is designated as a GMO and might be labeled as such in the exporting country. As a result, from the exporter’s point of view, this would create a non-tariff barrier problem.

Second, since there will be limits to how individual countries can deal with such situations, international frameworks or coordinated responses are necessary. Although it would be desirable to have a database registration of products and an identification code for each product, such as an equivalent to the Biosafety Clearing House and unique identifiers in GMOs, it would be difficult to achieve.^29^ Efforts to form international governance for genome-edited organisms are not discussed frequently at the CPB, the Codex and the OECD, and it would be difficult to formulate international rules to avoid confusion over such imports and exports in the short term. As Jasanoff and Hurlbut (2018) pointed out, in addition to issues related to trade, international discussion on ethical and other issues related to genome-editing technologies is crucial. The pressing future task is to establish a framework, such as global observatory, to facilitate international dialogue on various issues.

Third, as a kind of international response to the above, the same policy could be introduced in regions with close trade interdependence. For example, since Argentina decided on its policy in 2015, Brazil, Chile, and Colombia have introduced almost identical rules (Kuiken and Kuzma, 2021). All these South American countries adopt Approach 3 and other countries in this region would follow suit. In addition to the South American countries, the United States, Australia, and others have also published a communication in the World Trade Organization (WTO), claiming that differences in the rules surrounding genome-editing technologies could impede trade and innovation (WTO Committee on Sanitary and Phytosanitary Measures, 2018).

**Table 4** shows expected response of importing country in different situations.

## 4 Conclusion

Scientific factors alone cannot explain the emergence of the abovementioned convergence phenomenon in the formation of rules concerning genome-edited organisms. This study identifies four approaches to the regulatory consideration of genome-edited organisms in various countries and demonstrates that these approaches are converging into two main approaches. The study then discusses the factors that have led to this convergence, using the concepts of regulatory competition and organizational similarity, and the underlying factors of this convergence, focusing on the fact that there have been no major negative responses to genome-editing technology. Even though genome-editing technology has attracted widespread attention as a game changer in the life sciences, it is important to point out that no country has revised its basic laws on genetic modification. Therefore, convergence in a strict sense, has not been reached. Furthermore, this study discusses issues regarding the challenges posed by the situation of two separate approaches, particularly from the perspective of trade and other issues. Further convergence would require a revision of the basic legislation, and there is no guarantee that the initially intended objectives would be achieved by inviting a political process. In this sense, further convergence is difficult to foresee in the short term.

In the international perspective, many countries have not yet completed their policy-making processes. As these countries move forward, patterns other than those discussed in this paper may emerge, or further convergence may occur. Of particular importance is the possibility that the policy convergence observed in this study may be reversed as a result of a major civil society resistance against genome-editing technologies triggered by trade disruption or other factors. In this sense, the discussion in this paper needs to be further explored. In addition, this study did not examine in detail who participated in the policy-making process in the administrative bodies. Depending on who participates, the detailed design of the rules (e.g., disclosure of notified information) and the time it takes to formulate the rules may vary. If a trade issue arises and becomes the cause of a dispute in the World Trade Organization, the dynamics may take on a different dimension than the policy convergence observed in this paper. Policy competition can lead to convergence to a certain extent, but, as pointed out above, it is far from convergence at the level of basic GMO legislation. To get there, dynamics at a different level from policy competition might be needed. This issue, however, is beyond the scope of this paper. Nevertheless, we need to continue to monitor policy trends closely.

## Author contributions

MT and MM discussed the study design and conducted research reviews and interviews. MT drafted the manuscript based on the study and MM edited the manuscript. All authors contributed to the article and approved the submitted version.

## Footnotes

1 This paper discusses 10 countries/regions, ranging from Europe (EU) to North America (US) to South America (Argentina) to Oceania (Australia and New Zealand) to Asia (Japan, China, India, and the Philippines). These countries and regions were selected because they are considered suggestive of the diversity of rules surrounding genome-editing technology at this point in time. Other countries are also mentioned in Table 2 to present the status of rulemaking.

2 According to Podevin et al. (2013: 376-77), three types SDNs are explained as follows. “The SDN-1 techniques use SDNs to generate site-specific random mutations; in most cases via a single DSB [double-strand breaks] that plants repair mainly by nonhomologous end joining (NHEJ).” “The SDN-2 techniques are based on the use of an externally supplied DNA template for the repair that proceeds by homologous recombination (HR).” “[T]he SDN-3 techniques aim to introduce long DNA fragments (e.g., transgenes) at a predefined locus using a donor DNA […] in combination with an SDN. This facilitates the targeted integration of DNA […].”

3 In the UK, a new category of organism called “precision bred organisms” is being proposed which “will be subject to a new regulatory framework as defined in the Genetic Technology Bill. This framework will contain aspects of both the GMO legislative framework, which will remain in place for all other organisms made using modern biotechnology, and the framework as it applies to traditionally bred organisms. As such, the level of regulatory scrutiny is somewhere between that of GMOs and traditionally bred organisms.” (UK Parliament Impact Assessment 2022, p.20) https://publications.parliament.uk/pa/bills/cbill/58-03/0011/ImpactAssessmentGeneticTechnology(PrecisionBreeding)Bill.pdf

4 The Coordinated Framework has been updated in 1992 and 2017.

5 85 Fed. Reg. 29790 (May 18, 2020)

6 In addition, state governments also have authority for oversight of transgenic crops and collaborate with federal agencies. In the case of state government oversight, interests exist not only in health and the environment but also from an economic perspective (Taylor et al. 2004).

7 Whether to allow developers to self-determine is one of the key issues in the SECURE regulations, and its pros and cons are discussed in detail in the Federal Register (85 Fed Reg 29798, May 18, 2020).

8 However, developers can request a confirmation from the USDA through the “Confirmation Request Process” that their product is not subject to regulation. The result, that is, the confirmation letter, will be posted on the USDA website. Since US companies have product liability for food, they are also likely to be eager to obtain safety confirmation from the government.

9 85 Fed. Reg. 64308 (October 9, 2020)

10 https://www.fda.gov/news-events/press-announcements/fda-makes-low-risk-determination-marketing-products-genome-edited-beef-cattle-after-safety-review

11 With regard to transgenic Glofish, the FDA also exercised enforcement discretions on the grounds that they would pose low risk. However, such exercise of discretionary authority is a decision that is made only after an application for approval has been filed, and there is little predictability on the part of the applicant.

12 As Kuiken and Kuzma, 2021 point out, Argentina was the first country in the South American region to adopt the rule, and other countries, such as Brazil, Chile, Colombia, Paraguay, Honduras, and Guatemala have followed suit. Therefore, the Argentine example is used here to represent it.

13 In Brazil, however, limited information of notification to the government will be published in the Federal Gazette, according to the Normative Resolution No.16 (January 15, 2018) by the National Biosafety Technical Commission (CTNbio).

14 Another type, SDN-3, is treated as a genetically modified organism not only in Australia but also internationally because of the introduction of a foreign gene into its DNA.

15 https://www.foodstandards.gov.au/code/proposals/Documents/P1055%201st%20Call%20for%20Submissions.pdf

16 Please refer to the USDA-FAS website as follows. https://www.fas.usda.gov/data/china-mara-issues-first-ever-gene-editing-guidelines

17 For example, the Chinese government recently revised the biosafety assessment from being on a “crop variety and event” basis to solely on an “event” basis. English explanations can be found in the following report. https://apps.fas.usda.gov/newgainapi/api/Report/DownloadReportByFileName?fileName=Agriculture%20GMOs%20Safety%20Assessment%20Administrative%20Measures%20Finalized_Beijing_China%20-%20People%27s%20Republic%20of_01-24-2022.pdf

18 Office Memorandum F. No. C-12013/3/2020-CS-III, https://dbtindia.gov.in/sites/default/files/Final_%2011052022_Annexure-I%2C%20Genome_Edited_Plants_2022_Hyperlink.pdf

19 https://dbtindia.gov.in/sites/default/files/Final_%2011052022_Annexure-I%2C%20Genome_Edited_Plants_2022_Hyperlink.pdf

20 https://www.da.gov.ph/wp-content/uploads/2022/06/mc08_s2022_Revised.pdf

21 Regarding whether regulatory convergence is occurring, this paper focuses mainly on (1) whether the product is categorized as a GMO or (2) whether notification is required. It does not focus on other aspects, such as the meaning of SDN-2 (Jones et al., 2022) or whether notification results should be made public. In this sense, there are some aspects that are not fully converged in the strict sense of the word.

22 South Africa has decided in October 2021 to follow the same policy as the EU regarding new breeding techniques. This is presumably because the definition of GMO in South Africa is largely in line with the EU’s Directive, making it difficult to introduce different regulations. For more information, please refer to the following website. https://acbio.org.za/gm-biosafety/battle-regulation-new-breeding-techniques-south-africa/#:~:text=In%20October%202021%2C%20the%20South,of%20new%20genetic%20engineering%20technologies

23 Opportunities have been taken to exchange information, such as the workshop or conference held in February 2014, June 2018, but there has been no significant activity toward policy harmonization among member countries. For example, the role played by the OECD in the process of discussion for unconfined use of GM organisms (such as the publication of the so-called Blue Book in 1986) has not been fulfilled this time.

24 In other words, it is difficult to analyze the policy regarding genome-editing technology as a mere transfer or diffusion of policy because the policies regarding GMOs in each country differ in their starting points. However, if a particular regulatory model is diffused in a region with similar GMO policies as a starting point (e.g., South America), it may be possible to use policy transfer theory to analyze the diffusion of the model.

25 This point may be explained in terms of organizational isomorphism, as discussed by DiMaggio and Powell (1991). It is thought that administrative organizations in charge of regulating genetic modification originally had organizational isomorphism across countries, and that the probability of forming similar rules increased as a result of these administrative organizations considering rules without political processes.

26 Conversely, social recognition of such movements is limited in the US, as social movement organizations that oppose to genetic modification tend to pursue legal battles. This kind of difference between the EU and the US regarding the form of protest movement can be explained by each jurisdiction’s preference of rulemaking, as discussed by Bradford (2020, 41): “The EU’s tendency to respond to various regulatory risks with stringent standards partially stems from its reliance on ex ante government regulation as opposed to ex post enforcement by private litigants.” This contrast might be rooted in the different legal traditions between civil law and common law. Because of this backdrop, even if commercialization of genome-edited products in the US results in filing lawsuits against industry, this kind of social movement tactic tends to be less recognized by the general public.

27 Public trust has been pointed out as a factor influencing European NGOs’ criticism of GMOs (Aerni and Bernauer, 2006). The discourse that European NGOs have won public trust by criticizing GMOs, serving their corporate interests, has influenced NGOs abroad and resulted in a polarization of opinion for and against GMOs. If EU policy shifts and NGOs develop this discourse on genome-edited products, the impact may spread abroad. In this case, divergence, not convergence, could occur.

28 For example, GM Watch’s argument can be found at the following website. https://www.gmwatch.org/en/106-news/latest-news/20090-gene-editing-just-label-it

29 The development of detection techniques also faces difficulties in reality (European Network of GMO Laboratories, 2019).
